# Duplicated Paralogous Genes Subject to Positive Selection in the Genome of *Trypanosoma brucei*

**DOI:** 10.1371/journal.pone.0002295

**Published:** 2008-05-28

**Authors:** Richard D. Emes, Ziheng Yang

**Affiliations:** 1 Institute for Science and Technology in Medicine, Keele University, Stoke-on-Trent, United Kingdom; 2 Department of Biology, University College London, London, United Kingdom; University of Texas Arlington, United States of America

## Abstract

**Background:**

Whole genome studies have highlighted duplicated genes as important substrates for adaptive evolution. We have investigated adaptive evolution in this class of genes in the human parasite *Trypanosoma brucei*, as indicated by the ratio of non-synonymous (amino-acid changing) to synonymous (amino acid retaining) nucleotide substitution rates.

**Methodology/Principal Findings:**

We have identified duplicated genes that are most rapidly evolving in this important human parasite. This is the first attempt to investigate adaptive evolution in this species at the codon level. We identify 109 genes within 23 clusters of paralogous gene expansions to be subject to positive selection.

**Conclusions/Significance:**

Genes identified include surface antigens in both the mammalian and insect host life cycle stage suggesting that competitive interaction is not solely with the adaptive immune system of the mammalian host. Also surface transporters related to drug resistance and genes related to developmental progression are detected. We discuss how adaptive evolution of these genes may highlight lineage specific processes essential for parasite survival. We also discuss the implications of adaptive evolution of these targets for parasite biology and control.

## Introduction

Sub species of the parasite, *Trypanosoma brucei* from the family trypanasomatidae are the causative agent of Nagana in livestock and human sleeping sickness. No vaccines exist for this disease and current control regimes face problems of emerging drug resistance and toxicity [1]. This economic and medical importance of *Trypanosoma* species promoted whole genome sequencing of *T. brucei* and continued efforts to interpret the genome of this organism [2]. Through this genome project it was hoped that the accumulation and interpretation of data would provide an opportunity to better understand trypanosome biology, and hence improve disease control by identification of potential new drug targets, and by greater understanding of resistance to current drug control strategies. Whole genome data can facilitate investigation of a particular trait or disease if candidate genes are known *a priori*, or they can be utilised to search globally for extraordinary evolution and adaptation of genes which may reveal novel insights to species-specific biology.

To this end, we have inferred natural selection by estimation of *ω*, the ratio of non-synonymous (*d*_N_, amino acid changing) to synonymous (*d*_S,_ amino acid retaining) substitution rates (*ω* = *d*_N_/*d*_S_). With *ω*<0, *ω* = 1 and *ω*>1 representing purifying, neutral and adaptive evolution, respectively [3]. Identification of genes whose *ω* ratio is greater than 1 is thus persuasive evidence for adaptive evolution of the gene [4]. The validity of this type of approach has been verified both by computer simulations [5], [6] and by a growing number of cases, including recent reports of experimental verification of statistical predictions (for review see [7]).

**Figure 1:**
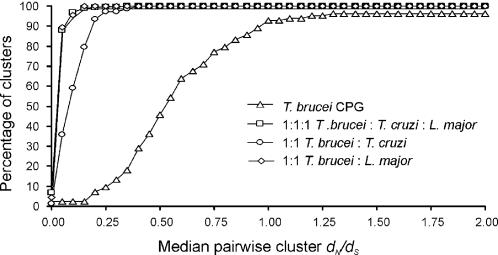
*d*_N_/*d*_S_ values for CPG and COG genes. Cumulative frequency plot of *d*_N_/*d*_S_ values for duplicated paralogous *T. brucei* CPGs in comparison to *T. brucei-T. cruzi* and *T. brucei-L. major* COGs. The *T. brucei* CPGs exhibit a relaxed purifying selection and are more likely to be subject to positive adaptive evolution.

Previous analysis of the *T. brucei* genome together with related trypanasomatidae (*T. cruzi* and *Leishmania major*) identified families of orthologous genes shared between these species and gene families which are specific to the *T. brucei* lineage [8]. Sadly, the estimated long divergence time (200–500 MY) [9], [10] between these three species precludes the confident interpretation of methods to model adaptive evolution across the tri-genome orthologous gene sets. However, using pairwise comparisons of genes within these groups we can show that for the majority of orthologous genes *ω* is small, confirming the general assumption that non-synonymous mutations are selected against and that purifying selection is the dominant force in evolution. In contrast, paralogous gene expansions in *T. brucei* exhibit a relaxation of selection and are more likely to be subject to positive selection (Figure 1). Thus we have focused our studies on the adaptive evolution of the duplicated paralogous gene families in the single parasite *T. brucei*.

We identify lineage specific genes evolving by duplication and adaptive evolution which are surface proteins expressed in both the insect vector and mammalian hosts, and proteins essential for development, and survival of the parasite.

## Results

### Comparison of pairwise estimates of selection

The clusters of homologous *T. brucei, T. cruzi* and *L. major* genes identified previously [8] were separated into four groups; clusters of orthologous genes (COGs) of 1:1:1 orthologous trios without duplication in any lineage (1174 clusters); COGs of 1:1 *T. brucei*:*T. cruzi* gene pairs (67 clusters); 1:1 *T. brucei*:*L. major* gene pairs (78 clusters) or *T. brucei* clusters of paralogous genes (CPGs, 90 clusters). Within each cluster every gene was compared in a pairwise manner and “gene-wide” estimates of *d*_N_/d_S_ were determined. A median value for each cluster was calculated. Where estimates from codeml are at the upper bound (999 for *d*_N_/d_S_) the values were recorded as infinity and were not included in median calculations.

A cumulative percentage frequency plot of the median cluster *d*_N_/d_S_ shows that for the majority of genes, comparisons between species typically exhibit strong purifying selection (Figure 1). Conversely the *T. brucei* CPG distribution shows a definite shift to the right representing a relaxation of purifying selection and a higher percentage of genes with *d*_N_/d_S_>1 (Figure 1). As lineage specific duplicates are often found to be the subject to adaptive evolution [11]–[13] these genes offer and exciting insight into the specific biology of *T. brucei* and were investigated further.

### Duplicated genes subject to positive selection

Of the 90 *T. brucei* CPGs, 40 contained three or more genes and could be investigated for adaptive evolution using codon models (see methods). Using this approach 23 CPGs containing a total of 109 genes showed robust evidence of adaptive evolution at *p*<0.05 in both M2 and M8 tests (1 cluster with p<0.05, 3 clusters with *p*<0.01 and 28 with *p*<0.001 in both M1–M2 and M7–M8 tests, Table 1). The Benjamini-Hochberg method [14] for controlling the false discovery rate in multiple comparisons was implemented at the α = 0.05 level. A single significant gene cluster (procyclin precursor) was non-significant following this method of correction. However using an additional stringent test of selection (M8a) all 23 CPGs including the procyclin CPG showed significant evidence of positive selection at *p*<0.05 following Benjamini-Hochberg correction, thus we consider these 23 CPGs to have significant evidence of positive selection. Parameter estimates for each positively selected cluster are shown in Supplementary Table S1. The high proportion of CPGs with evidence of positive selection (23/40, 57.5%) supports the assumptions that paralogous gene expansions often form the substrate for adaptive change. Additionally, it is evident that multiple clusters of related genes were identified as being subject to adaptive evolution, highlighting both the extensive gene duplication within these families consistent with previous studies [15] and the impact of adaptive evolution within particular gene types (Table 1).

**Table 1:** Summary of sites tests for positive selection.

Description	CPG^a^	N	LRT statistics			Parameter estimates		
			M1 vs. M2	M7 vs. M8	M8 vs. M8a	% of sites with d_N_/d_S_>1		d_N_/d_S_
						M2	M8	
65 kDa ISG	19416372	3	13.77^**^	14.58^***^	13.77^***^	8	8	4.34
65 kDa ISG	21719250	5	45.50^***^	45.56^***^	45.49^***^	12	12	12.68
75 kDa ISG	20195704	3	40.73^***^	41.14^***^	40.73^***^	18	18	3.96
Adenosine transporter	20117965	6	24.49^***^	29.03^***^	22.74^***^	1	1	9.56
Amino acid transporter	22063411	3	23.20^***^	23.24^***^	23.20^***^	11	11	8.14
Amino acid transporter	19796418	3	30.47^***^	30.52^***^	30.51^***^	3	3	27.7
Procyclin	19416463	5	6.11^*^	8.18^*^	6.10^*^	1	1	8.24
GRESAG2	20975481	3	18.36^***^	18.51^***^	18.36^***^	5	5	17.69
Hypothetical	20115952	3	10.89^**^	10.87^**^	10.24^**^	11	15	10.07
Hypothetical	19340333	4	13.43^**^	13.43^**^	13.43^***^	6	6	10.72
Hypothetical	20206217	4	13.49^**^	13.51^**^	13.49^***^	4	4	23.94
Hypothetical	21586974	9	16.62^***^	16.63^***^	16.08^***^	14	14	5.86
Hypothetical	21769349	3	16.18^***^	16.49^***^	16.18^***^	9	9	10.6
Receptor-type AC	19651441	5	46.95^***^	47.02^***^	46.95^***^	9	9	7.21
Receptor-type AC	20115358	8	131.60^***^	131.41^***^	131.09^***^	7	7	4.6
Receptor-type AC	21943524	8	183.29^***^	182.30^***^	179.44^***^	6	5	6.06
Receptor-type AC	21139502	5	279.88^***^	279.75^***^	279.74^***^	10	10	12.95
RHS	20343633	6	17.58^***^	17.89^***^	17.56^***^	22	25	2.33
RHS	20439293	3	32.94^***^	33.07^***^	32.94^***^	9	9	12.26
RHS	20461581	5	38.14^***^	38.36^***^	38.09^***^	3	3	35.12
RHS	19651414	8	102.43^***^	102.55^***^	102.43^***^	18	18	6.87
RHS	21995461	3	106.01^***^	106.08^***^	105.98^***^	4	4	33.46
RHS	20529562	4	157.08^***^	157.19^***^	156.87^***^	7	7	10.12

N, number of species analysed; M1 vs. M2, likelihood ratio test statistic for model M1 versus M2; M7 vs. M8, likelihood ratio test statistic for model M7 versus M8; Parameter estimates: percentage of sites in dN/dS>1 category and estimated dN/dS parameter under model M8. ISG, invariant surface glycoprotein. RHS retrotransposon hot spot protein. ^*^ Significance with P<0.05; ^**^ Significance with P<0.01; ^***^ Significance with P<0.001. A CPG jaccard cluster number from El Sayed et al [8].

### Functional annotation of positively selected genes

**Figure 2:**
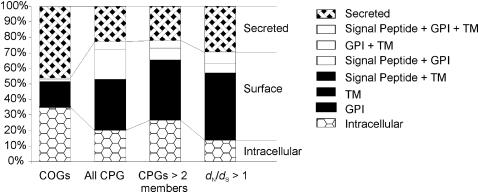
Histogram of predicted location of COG and CPG genes. Protein products were tested for transmembrane (TM) domains, secretory signal sequences and glycosylphosphatidylinositol (GPI) anchor sequences. Secreted; secretory signal sequence positive TM and GPI-anchor negative, surface; TM or GPI-anchor positive, Intracellular; secretory signal, TM and GPI-anchor negative. COGs, all 1:1:1 *T. brucei*:*T. cruzi:L. major* clusters if orthologous genes. All CPG, all *T. brucei* CPGs. CPGs>2 members, all CPGs tested for positive selection using codon model. *d*_N_/*d*_S_>1, CPGs subject to adaptive evolution using codon models.

Annotation of the predicted cellular location of encoded proteins suggests that genes at the surface or secreted from the parasite are more often subject to adaptive evolution, (Table 2). Further investigation of all genes highlights that whilst compared to the COGs those genes found within CPGs are more often predicted to be surface-located either through possession of transmembrane or GPI anchored regions (Figure 2). However, only the secreted category is significantly over abundant (*p* = 0.044 by binomial test).

**Table 2:** Functional annotation of CPGs subject to positive selection.

Description	CPGs	Predicted Location	Size ^a^	Pfam Domains	Biological Process	Molecular Function
65 kDa ISG	19416372, 21719250	Su	434–436	n/d	n/d	n/d
75 kDa ISG	20195704	Su	522–523	n/d	n/d	n/d
Adenosine transporter	20117965	Su	462–466	MFS_1, Nucleoside_tran	transport	nucleoside transporter activity
Amino acid transporter	19796418, 22063411	Su	450–490	Aa_trans, Trp_Tyr_perm	amino acid transport	amine transmembrane transporter activity
EP1 procyclin	19416463	Su	123–141	n/d	n/d	n/d
GRESAG2	20975481	Su	454–457	n/d	n/d	n/d
Receptor-type AC	19651441, 20115358, 21139502, 21943524	Su, Se	1170–1288	Guanylate_cyc	intracellular signalling cascade, cyclic nucleotide biosynthetic process	phosphorus-oxygen lyase activity
RHS	19651414, 20343633, 20439293, 20461581, 20529562, 21995461	Se, Su, In	557–860	n/d	n/d	n/d

Predicted cellular location based on GPI signal peptide and transmembrane domain prediction for each member of each cluster (see methods) Su; surface, Se; secreted, In; Intracellular. Pfam domains were detected using HMMER [47] to search the Pfam database [54]. Gene Ontology terms were linked to predicted Pfam domains using Pfam2GO (http://www.geneontology.org/). For details of gene annotation and prediction of location see methods.

Analysis of Pfam domains and gene ontology (GO) terms showed that the positively selected genes represent a small functional group of proteins, with only five Pfam domain types and associated GO terms detected. Although few in number, the domains detected are often specific or enriched in the CPGs compared to COGs and often in positively selected genes, for example 33 Guanylate cyclase domain containing proteins are detected from the 300 CPGs and 26 of these 33 are encoded by genes subject to positive selection, whilst only 14 Guanylate cyclase domains are found amongst the 4896 COG proteins. Additionally the Pfam domain Nucleoside_tran associated with nucleotide transport is only present in genes in CPGs and that all six proteins with this domain are under positive selection.

### Adaptive evolution of 65 KDa and 75 KDa invariant surface glycoproteins

Like the widely known variable surface glycoprotein (VSG) genes involved in parasite survival by antigen switching [16], the invariant surface glycoproteins (ISG65, ISG75) are also found at high density on the surface of the blood-stage form of the parasite [17]. The two forms of invariant proteins are distinguishable by mass although a single round of a PSI-BLAST search [18] identifies them as potential homologues (E value 3 ×10^−10^). Additionally, the conserved protein architecture of a large extracellular domain linked to a short intracellular domain by a single transmembrane domain betrays the likely common ancestry of both glycoprotein families.

Two clusters of ISG65 and a single cluster of ISG75 genes were identified to be evolving under positive selection. Of the ISG65 genes for the cluster 21719250 one codon was predicted to have *d*_N_/*d*_S_>1 and three codons for cluster 19416372 by both M2 and M8 models. In contrast 16 codons were predicted by M2 and M8 in the ISG75 cluster 20195704 (see table S1 for details of sites). Although none of the positions identified in either ISG65 or ISG75 are orthologous, all are located in the extracellular region.

### Adaptive evolution of adenosine transporters

The CPG 20117965 encodes the P1 form of the nucleoside transporter a predicted eleven transmembrane transporter protein. The four sites with significant evidence of *d*_N_/*d*_S_>1 in both the M2 and M8 models are located in the extracellular (EC) loops of this protein, one S55 in EC1 and three S333, M334 and F339 in EC4. The adenosine transporter 2 gene is part of the nucleoside transporter family and is of particular interest as loss of function of the paralogous TbAT1 gene which encodes the P2 type of nucleoside transporter is related to drug resistance in T. *brucei brucei* [19], [20]. However, sites with significant evidence of *d*_N_/*d*_S_>1 are not homologous to those mutations (L71V, A178T, G181E, D239G, N276S) seen in drug resistant isolates [21].

### Adaptive evolution of amino acid transporters

Two clusters of homologous amino acid transporter genes were identified as having evolved under positive selection, CPGs 19796418 and 22063411. These CPGs exhibited six and nine codons with significant evidence of adaptive evolution respectively. Recent analysis of the amino acid transporter genes in kinetoplastid species grouped these two clusters as a single *T. brucei* specific locus containing six genes (named AAT4Tb) which have potentially evolved by tandem duplication and which exhibit evidence of elevated evolutionary rate [22]. Here we show that each of the two clades of the AAT4Tb cluster exhibits evidence of positive selection. However, the sites detected are not orthologous between the two CPGs.

### Adaptive evolution of procyclin

A single cluster (CPG 19416463) of five procyclin genes exhibited positive selection, only a single site, G51 was predicted with *d*_N_/*d*_S_>1. The function of site G51 is unknown, but is N-terminal to the Glu-Pro repeat region. The number of Glu-Pro repeats and similarity searches places this procyclin in the EP3 procyclin family [23].

### Adaptive evolution of receptor-type adenylate cyclase genes

Four CPGs of receptor-type adenylate cyclase genes (ACs) were identified, containing a total of 29 genes. Like membrane bound cyclases of metazoans, trypanosome ACs are single transmembrane spanning proteins with an intracellular cyclase domain which has been crystallised (PDB 1FX2/1FX4 [24], [25]) to which each cluster was aligned.

**Figure 3:**
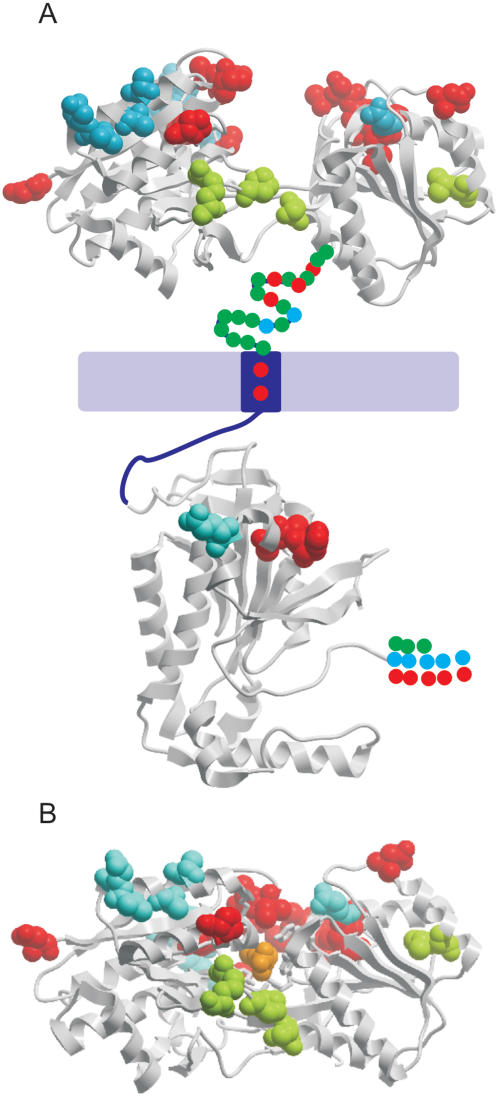
Codon-specific analysis of receptor-type adenylate cyclase genes. Homologous extracellular codons with predicted *ω*>1 are mapped to the tertiary structure of *E. coli* leucine-binding protein (PDB 2LBP [28]) or L-leucine-binding protein with leucine bound (PDB 1USK [56]). Intracellular codons that are predicted to be under positive selection are mapped to the tertiary structure of *T. brucei* receptor-type adenylate cyclise (PDB 1FX2 [25]). A) Hypothetical representation of the intact Trypanosome adenylate cyclase molecule. The extracellular region is composed of an N-terminal region homologous to 2LBP and a region of unknown function. A single transmembrane region links to the conserved C-terminal catalytic domain. Positively selected sites (posterior probability ≥0.95) homologous to crystal structures are shown as space filled residues. Red, CPG 21943524. Blue, 20115358. Green, 19651441. Positively selected sites in regions not homologous to a known crystal structure are shown as coloured circles. B) Position of positively selected sites following conformation change of extracellular binding region upon binding to leucine (coloured orange). Orientation is as for part A.

Predicted positively selected sites were largely located within the extracellular part of the protein, with only four codons predicted to be subject to positive selection in the intracellular cyclase domain (Figure 3). Previously the extracellular domain has been thought to have no similarity to other proteins or protein domains but is predicted to have a ligand binding role [26]. However a psi-blast search of the nr database with the n-terminal portion of a representative AC protein (Tb927.6.760) as a query identified a probable leucine/isoleucine/valine-binding protein precursor from the bacteria Bradyrhizobium japonicum (NP_773188.1 Psi-blast round 2 Evalue = 8×10^−10^) as a candidate homologue of the kinetoplastid AC family. To confirm this prediction, a hidden Markov model (HMM) of the n-terminal region of ACs was used to search HMMs generated from all PDB files (PDB version 70, April 2007) using HHpred a method of hidden Markov model comparison tool utilising secondary structure information to identify distant homologues with high sensitivity [27]. Using the local alignment mode, Tb927.6.760 aligned to *E. coli* L-leucine-binding protein (PDB 2LBP [28]) with a probability of 99.7 (Evalue 1.6×10^−14^).

## Discussion

Parasite genome sequencing projects provide an invaluable resource for biologists; however the successful mining of any large scale data set is key to new avenues of research. Thus a greater understanding of adaptive evolution within parasitic species will link genome studies to the biology of parasites and identify potential new targets for intervention. Here we have used the data from the genomes of human pathogens to scan for genes subject to adaptive molecular evolution and highlight the areas of the protein coding genome which have been extensively modified by natural selection. The genes identified are frequently duplicated, often independently in *T. brucei* and the majority of the expanded adaptive proteins are surface expressed, suggesting interaction with the external host environment.

Genes identified include adenosine transporters related to the P2 type associated with drug resistance [19], [20] and molecules involved in life cycle progression; the procyclins expressed in late procyclic parasites in the insect host [29], and Adenylate cyclises (ACs) which form part of the signal transduction pathway generating cAMP. ACs are the subject of particular interest as cAMP is thought to influence the life-cycle progression of *T. brucei* [24], and understanding of ACs may provide insight into the mechanism of parasite development and transmission. The extensive duplication and diversification of the ACs suggest that these genes may be part of an essential sensory system and exemplify the complexity of the control of parasite lifecycle progression. The migration and development in different hosts requires tight coupling of environmental sensing, gene expression and parasite development. The variation of the extracellular LBP homologous region of ACs could therefore be to detect multiple ligands by variation of binding specificity or association/dissociation dynamics of ligand interaction thus allowing a range of stimuli to potentiate a signal via the ACs. For example, in *T. cruzi* components of mammalian serum and cAMP are known to stimulate differentiation of proliferative epimastigotes to infective metacyclic trypomastigotes via adenylate cyclases [30], [31]. Fraidenraich *et al* 1993 showed that adenylate cyclase can bind α-globin peptide in the insect vector gut following a blood meal. They postulated that “*T. cruzi* could have several receptors with different specificity for globin derived peptides, or alternatively, only one receptor specific for a common domain shared by several α-globin chain species” [31]. Our results support the former hypothesis that positive selection has driven the adaptive evolution of the extracellular domain whilst functional constraint retains the intracellular domain by purifying selection.

This proposal is supported by experiments which demonstrate that in the LBP amide receptor of *P. aeruginosa*, mutation of Thr-Asn at position 106 alters ligand specificity, but that this is due to a change in conformation rather than T106 binding the ligand directly [32]. However, not all changes may alter function. For example, it has been shown that adaptive changes in the homologous AmiC ligand binding protein of *P. aeruginosa* which affect ligand specificity result in an unstable protein [33]. Therefore only a subset of mutations may actually alter substrate binding specificity or dynamics, whilst others may be compensatory to maintain structural stability. The number of positively selected sites residing in the 2LBP homologous region varies between clusters from 0 in cluster 21139502 to 17 in cluster 21943524, but in all clusters a greater number of positively selected sites were detected in the receptor 2LBP like portion compared to the catalytic 1FX4 domain. When mapped onto the 2LBP sequence, the sites are dispersed between the two lobes of the clam shell-like structure. Which come into close proximity to the ligand after binding and closing of the clam shell, (Figure 3b). Additionally a large number of sites are located in the region of unpredicted function between the 2LBP homologous region and the predicted TM domain.

### Adaptive Evolution: Implications for Parasite Biology

The impact of adaptive evolution on parasite biology stems from the premise that the fixation of duplicated genes is an adaptive event; that these duplicated genes act as a source for protein subfunctionalisation [12] by evolution of key positions in the protein, and that this adaptation is reflective of organism-specific selective pressures.

Importantly, the availability and continued understanding of a gene and genome evolution allows the rational design of experiments to assess function of positively evolving or extended gene families. If a gene of interest is part of a gene family, experimental procedures may result in little or unexpected results. For example, gene knockout is a powerful tool, but may not remove a phenotype if only part of the complement of a gene family is removed, e.g. McGwire *et. al*. [34] noted that the knockout of a single GP63 gene in Leishmania did not completely attenuate the parasite migration through the extracellular matrix and where removal of seven GP63 genes was required to reduce infectivity of *L. major* [35]. This residual enzymatic action can be explained by subfunctionalization of the duplicated genes. Thus experiments should be targeted to identify more subtle effects or should incorporate the genomic information such as the knockout of multiple genes or clusters of duplicated genes.

### Adaptive Evolution: Implications for Parasite Control

Selection of a protein as a target for therapeutics or for vaccine design follows some basic tenets; drug targets should ideally be pathogen specific genes/pathways so as to avoid affecting the individual being treated, and vaccine targets should be surface located to be accessible by the primed host immune system. With available genome data one can rapidly screen for genes which encode proteins fitting the above criteria. However, this study highlights that gene duplication and adaptive evolution should be considered during screening for potential targets. With regard to drug therapy, an anti-parasitic drug should be designed differently depending on whether it is to bind a single protein or to bind all proteins of a multi-gene family. Thus therapies which target one or a subset of duplicate genes may not be effective, at low doses due to binding affinities to variable targets. In this case, it may be more effective to target a single-copy gene, where removal of function should offer more complete perturbation of a pathway and hence more tractable control. Alternatively a drug would need to target a conserved region of the protein or have large effects at low affinity when binding to variable members of a protein family.

With respect to parasite vaccine design, it seems logical as proposed in the context of bacterial and virus vaccine design, that genes or gene regions undergoing adaptive evolution should be avoided when considering drug targets [36], [37]. This is exemplified by the long term success of poliovirus vaccines, which has been related to purifying selection maintaining the sequence and hence structure of targeted surface proteins [38] conversely targeting the rapidly evolving proteins of HIV may have contributed to developing resistance [39]. However, the complexity of this argument is exemplified in current vaccine candidates for *Plasmodium falciparum* control, where the relative merits and problems of sequence variation in vaccine candidates are currently discussed [40]. Indeed, both conserved regions [41] and full length proteins [42] of known polymorphic merozoite surface protein 3 (MSP3) are under investigation as important vaccine candidates, and it has been reported that polymorphic regions may induce a stronger immune response [43].

The conflict within these arguments are that potential candidate genes which are surface expressed and elicit a strong immune response and hence are good vaccine candidates are also those most likely to be subject to adaptive evolution. We propose these adaptive genes may simply be more malleable by natural selection and thus be more likely to change in the future, especially under the strong selective pressure of choreographed therapeutic intervention.

Theoretically therefore the challenge for future design of vaccines is to identify parasite specific molecules which do not bear the signature of rapid adaptive change, or to target regions of proteins distant from rapidly evolving regions. However, the practicality of design and the biology of the immune response may require that variable sites in multiple gene families are necessary as targets. Hence, the availability of complete genome sequence data is central to this as it allows evolutionary analysis to be incorporated in vaccine design from inception, stimulating novel hypotheses relating to the biology of these parasites, and a greater understanding of pathogen genome evolution.

## Materials and Methods

### Data collection and manipulation

Predicted protein and cDNA sequences were obtained from the Sanger Institute ftp server (ftp.sanger.ac.uk/pub/databases/). Membership of gene families based on reciprocal blastp searches and single linkage clustering using a jaccard similarity coefficient was taken from table S1 of El-Sayed et al [8]. Using this approach 8080 *T. brucei* genes, not labelled as pseudogenes, were grouped into clusters of orthologous genes (COGs) with *T. cruzi* and *L. major* (6585 3-species and 571 2-species COGs). 924 genes were labelled as *T. brucei* specific. Of these 924, 624 were present as single copy genes and 300 were duplicated in the *T. brucei* lineage and formed what we refer to as clusters of paralogous genes (CPGs).

Sequences with less than 10 amino acids were removed and the remaining aligned using muscle [44]. Protein alignments were then parsed to remove poorly aligned regions using the Gblocks algorithm [45] with the following criteria; maximum number of contiguous non-conserved positions = 10, minimum length of a block = 5, gap positions allowed in all sequences. These parsed alignments were then used to construct a corresponding cDNA alignment. Initial phylogenetic trees were inferred by neighbor joining under the JC69 model [46].

To predict cellular location, protein products were tested for the presence of transmembrane (TM) domains using TMHMM Server v2.0 [47], secretory signal sequences using the Sigcleave prediction module [48] and glycosylphosphatidylinositol (GPI) anchor sequences using DGPI (http://129.194.185.165/dgpi/index_en.html). Sequences were annotated as secreted if they were secretory signal sequence positive but TM and GPI-anchor negative, surface expressed if TM or GPI-anchor positive and intracellular if secretory signal, TM and GPI-anchor negative. All sequences were compared to a library of Pfam HMMs (obtained from www.sanger.ac.uk/Software/Pfam/ftp.shtml on 27/06/2007) using HMMER [49].

### Detection of adaptive evolution

A “gene-wide” estimate of adaptive evolution was estimated by pairwise calculation of *d_N_/d_S_* between all members of a cluster. Additionally, as adaptive evolution is likely to act on a small subset of amino acid residues and hence averages of substitution rates across the gene may not strictly indicate positive selection [4] we scanned the CPGs with three or more members (40 CPGs, 171 genes) for adaptive evolution using a codon model. To achieve this, data are fitted to codon-based substitution models that allow *ω* to vary among sites, with the parameters of the model estimated using maximum likelihood [4]. The analysis was conducted using the CodeML application from the PAML package version 3.15 [50]. For each pair of nested models the log likelihood values are compared using the likelihood ratio test (LRT). If the model allowing positive selection fits the data significantly better, as judged by the LRT, positive selection is inferred [51]. In this study we used two pairs of models: M1 (neutral) versus M2 (selection) [52]; and M7 (beta) versus M8 (beta+*ω*) [3]. M1 allows two *ω* site classes with *ω*_0_<1 estimated from the data or *ω*_1_ = 1. Whilst M2 allows an additional *ω*_2_ value to be estimated from the data which may be >1. M7 fits *ω* to 10 site classes between 0 and 1 approximating a beta distribution and M8 adds an additional site class with an *ω* possibly >1, estimated from the data. Both M1-M2 and M7-M8 comparisons were performed with 2 degrees of freedom. To speed the likelihood iterations M0 was used to estimate branch lengths based on the topology of the neighbour joining trees and the estimates of branch lengths were used as initial values in estimations by other models. To ascertain convergence of the likelihood iterations multiple runs were conducted until the difference between two log likelihoods for each model were less than or equal to 0.01.

When a gene cluster shows a signature of adaptive evolution according to the LRTs, the empirical Bayes method [52], [53] was used to identify specific codons which reside within the site class of *ω*>1. Codons are identified as undergoing adaptive evolution if both tests are significant and if the posterior probability under both M2 and M8 models was ≥0.95.

Additionally, to stringently test for evidence of positive selection and to remove the potential identification of relaxed purifying selection, we conducted a comparison of M8 model (where a single class of sites is allowed with *d_N_/d_S_*>1) to M8a, where *d_N_/d_S_* = 1 [54]. The reliance of three M2 vs M3, M7 vs M8 and M8 vs M8a nested LRTs to infer positive selection also provides some protection against false positives identified as a result of potential recombination events [55].

All sequences predicted to be subject to positive selection were used to search for homologous sequences in the PDB database of protein structures (http://www.rcsb.org/pdb/ accessed August 2006) using BLAST [18]. Molsoft ICM browser (molsoft.com) was used for structural manipulations.

## Supporting Information

Table S1 Parameter estimates for all positively selected CPGs. Clusters of orthologous genes and descriptions from El-Sayed et al [8]. Parameter estimates predicted by PAML models M0, M1, M2, M7 and M8.
(0.19 MB DOC)
