# Supplementary material for: Duplicated Paralogous Genes Subject to Positive Selection in the Genome of Trypanosoma brucei
Source: PLoS One. 2008 May 28;3(5):e2295. doi: 10.1371/journal.pone.0002295 (PMC2386149; doi:10.1371/journal.pone.0002295)
Supplement: Table S1 — Parameter estimates for all positively selected CPGs. Clusters of orthologous genes and descriptions from El-Sayed et al [8]. Parameter estimates predicted by PAML models M0, M1, M2, M7 and M8. (0.19 MB DOC) [file pone.0002295.s001.doc]

| **19340333** | **p** | **lnL** | **Kappa** | **Estimates of Parameters** | **Positively Selected Sites** |
| --- | --- | --- | --- | --- | --- |
| **M0** | 7 | -1812.987312 | 2.50 | w = 0.658 | None |
| **M1** | 3 | -1807.308954 | 2.40 | p0 = 0.501 p1 = 0.499 | Not allowed |
| **M2** | 5 | -1800.595035 | 2.76 | p0 = 0.937 p1 = 0.000 p2 = 0.063 w2 = 10.718 | A132* Y134* S142* G146* E220* |
| **M7** | 3 | -1807.309034 | 2.40 | p = 0.005 q = 0.005 | Not Allowed |
| **M8** | 5 | -1800.595941 | 2.76 | p0 = 0.937 p1 = 0.063 p = 61.388 q = 99.000 w = 10.723 | A132* Y134* T137* S142* G146** E185* D217* E220* P237* |
| **19416372** | **p** | **lnL** | **Kappa** | **Estimates of Parameters** | **Positively Selected Sites** |
| **M0** | 5 | -2893.148613 | 2.13 | w = 0.502 | None |
| **M1** | 3 | -2866.809193 | 2.15 | p0 = 0.563 p1 = 0.437 | Not allowed |
| **M2** | 5 | -2859.926060 | 2.39 | p0 = 0.611 p1 = 0.314 p2 = 0.075 w2 = 4.389 | D203* Q225* Q227* |
| **M7** | 3 | -2867.216849 | 2.14 | p = 0.005 q = 0.007 | Not Allowed |
| **M8** | 5 | -2859.926836 | 2.39 | p0 = 0.922 p1 = 0.078 p = 0.056 q = 0.102 w = 4.338 | Y120* T171* D203** G224* Q225* Q227* |
| **19416463** | **p** | **lnL** | **Kappa** | **Estimates of Parameters** | **Positively Selected Sites** |
| **M0** | 9 | -781.271450 | 3.34 | w = 0.121 | None |
| **M1** | 3 | -777.574219 | 3.28 | p0 = 0.961 p1 = 0.039 | Not allowed |
| **M2** | 5 | -774.520865 | 3.30 | p0 = 0.993 p1 = 0.000 p2 = 0.007 w2 = 8.236 | G51* |
|  |  |  |  |  |  |
| **M7** | 3 | -778.644148 | 3.36 | p = 0.114 q = 0.763 | Not Allowed |
| **M8** | 5 | -774.552735 | 3.30 | p0 = 0.993 p1 = 0.007 p = 10.022 q = 99.000 w = 8.243 | G51* |
| **19651414** | **p** | **lnL** | **Kappa** | **Estimates of Parameters** | **Positively Selected Sites** |
| **M0** | 15 | -4423.588809 | 1.93 | w = 0.945 | None |
| **M1** | 3 | -4372.350963 | 1.56 | p0 = 0.613 p1 = 0.387 | Not allowed |
| **M2** | 5 | -4321.135537 | 2.07 | p0 = 0.751 p1 = 0.072 p2 = 0.177 w2 = 6.874 | R9** W120** D123* R124* S128* G134** G135* R158* V214** D215** R270* V277* A279** V284** R285** L293* E294** Q295* T296* L332* F351* G363* G365** C366* A370** E374** S380* H401* T403* S405** S446* S448* S449** H450* F452* M453* E519* A520* L537* |
| **M7** | 3 | -4372.413492 | 1.57 | p = 0.005 q = 0.008 | Not Allowed |
| **M8** | 5 | -4321.136922 | 2.07 | p0 = 0.823 p1 = 0.177 p = 0.157 q = 0.896 w = 6.870 | R9** W120** D123** R124* S128* G134** G135** R158* S170* V214** D215** R260* R270** V277* A279** G283* V284** R285** L293** E294** Q295** T296* L332* F351* G363* G365** C366* E369* A370** E374** S380* E396* H401* T403* S405** S446* S448* S449** H450** F452* M453* E519** A520** L537* |
| **19651441** | **p** | **lnL** | **Kappa** | **Estimates of Parameters** | **Positively Selected Sites** |
| **M0** | 9 | -6050.070093 | 3.22 | w = 0.532 | None |
| **M1** | 3 | -6030.846613 | 2.94 | p0 = 0.687 p1 = 0.313 | Not allowed |
| **M2** | 5 | -6007.371965 | 3.40 | p0 = 0.915 p1 = 0.000 p2 = 0.085 w2 = 7.214 | S169* W174* N186* A263* K466* Q482* K530* N532* K534* L536* F575* L609* S618* G647* I650* P688* N690* K727* K769* M1238* H1239* L1240* |
| **M7** | 3 | -6030.881666 | 2.91 | p = 0.005 q = 0.012 | Not Allowed |
| **M8** | 5 | -6007.371969 | 3.40 | p0 = 0.915 p1 = 0.085 p = 0.005 q = 1.907 w = 7.214 | S169* W174* N186* A263* K466* Q482* K530* N532* K534* L536* F575* L609* S618* G647* I650* P688* N690* K727* K769* M1238** H1239* L1240* |
| **19796418** | **p** | **lnL** | **Kappa** | **Estimates of Parameters** | **Positively Selected Sites** |
| **M0** | 5 | -2126.464229 | 1.60 | w = 0.421 | None |
| **M1** | 3 | -2117.447367 | 1.51 | p0 = 0.706 p1 = 0.294 | Not allowed |
| **M2** | 5 | -2102.214081 | 1.36 | p0 = 0.966 p1 = 0.000 p2 = 0.033 w2 = 26.454 | T266* V283* T286* W445** N449** I450* |
| **M7** | 3 | -2117.451278 | 1.52 | p = 0.005 q = 0.012 | Not Allowed |
| **M8** | 5 | -2102.191219 | 1.36 | p0 = 0.969 p1 = 0.031 p = 0.017 q = 0.091 w = 27.699 | T266* V283* T286* W445** N449** I450* |
| **20115358** | **p** | **lnL** | **Kappa** | **Estimates of Parameters** | **Positively Selected Sites** |
| **M0** | 15 | -10743.448970 | 2.41 | w = 0.547 | None |
| **M1** | 3 | -10535.264262 | 2.25 | p0 = 0.610 p1 = 0.390 | Not allowed |
| **M2** | 5 | -10469.464960 | 2.50 | p0 = 0.613 p1 = 0.320 p2 = 0.066 w2 = 4.806 | G95** H97** H104** G106** E285* E328* S334** T336** G337* Q338* D362* F615** E617** T828* R835** E1057* F1101* V1108* F1230* D1235** |
| **M7** | 3 | -10535.425750 | 2.26 | p = 0.005 q = 0.007 | Not Allowed |
| **M8** | 5 | -10469.719039 | 2.50 | p0 = 0.928 p1 = 0.072 p = 0.021 q = 0.040 w = 4.603 | K89* G95** H97** M98* T100* H104** G106** K107* N250* E285* E328** D331* S334** T336** G337** Q338** D362** Q610* F615** E617** G826* T828* F831* R835** G836* E1057* F1101** V1108* T1109* P1122* P1204* F1212* G1215* M1222* F1230* G1232* D1235** G1238* D1243* L1244* |
| **20115952** | **p** | **lnL** | **Kappa** | **Estimates of Parameters** | **Positively Selected Sites** |
| **M0** | 5 | -1638.669150 | 3.05 | w = 1.101 | None |
| **M1** | 3 | -1638.704834 | 3.00 | p0 = 0.443 p1 = 0.557 | Not allowed |
| **M2** | 5 | -1633.259177 | 3.24 | p0 = 0.888 p1 = 0.000 p2 = 0.112 w2 = 11.758 | D190* |
| **M7** | 3 | -1638.704834 | 3.00 | p = 9.708 q = 0.005 | Not Allowed |
| **M8** | 5 | -1633.271902 | 3.24 | p0 = 0.853 p1 = 0.147 p = 0.122 q = 6.817 w = 10.073 | L188* D190* |
| **20117965** | **p** | **lnL** | **Kappa** | **Estimates of Parameters** | **Positively Selected Sites** |
| **M0** | 11 | -4497.577794 | 2.15 | w = 0.239 | None |
| **M1** | 3 | -4391.110315 | 2.18 | p0 = 0.780 p1 = 0.220 | Not allowed |
| **M2** | 5 | -4378.866772 | 2.35 | p0 = 0.781 p1 = 0.213 p2 = 0.006 w2 = 11.654 | S55* S333** M334** F339* |
| **M7** | 3 | -4394.196104 | 2.15 | p = 0.109 q = 0.325 | Not Allowed |
| **M8** | 5 | -4379.683511 | 2.31 | p0 = 0.992 p1 = 0.008 p = 0.130 q = 0.417 w = 9.558 | S55* A237* S333** M334** F339* |
| **20195704** | **p** | **lnL** | **Kappa** | **Estimates of Parameters** | **Positively Selected Sites** |
| **M0** | 5 | -3333.269256 | 2.36 | w = 0.540 | None |
| **M1** | 3 | -3293.543692 | 2.15 | p0 = 0.625 p1 = 0.375 | Not allowed |
| **M2** | 5 | -3273.178581 | 2.64 | p0 = 0.816 p1 = 0.000 p2 = 0.184 w2 = 3.956 | Q35** G42* Q45* T52* E56* A75* Y76* G114* Y120* V156* L157* P158* S224* R275* F277** Y287** |
| **M7** | 3 | -3293.750068 | 2.18 | p = 0.005 q = 0.007 | Not Allowed |
| **M8** | 5 | -3273.179845 | 2.64 | p0 = 0.816 p1 = 0.184 p = 6.985 q = 99.000 w = 3.957 | Q35** G42** Q45* Q49* T52** E56* A75* Y76* L97* S101* G114* I118* Y120* G122* V156* L157* P158* G161* L167* V199* S224* A225* T271* R275** F277** Y287** A417* |
| **20206217** | **p** | **lnL** | **Kappa** | **Estimates of Parameters** | **Positively Selected Sites** |
| **M0** | 7 | -406.262943 | 8.51 | w = 1.078 | None |
| **M1** | 3 | -404.522554 | 6.96 | p0 = 0.569 p1 = 0.431 | Not allowed |
| **M2** | 5 | -397.778663 | 9.62 | p0 = 0.588 p1 = 0.372 p2 = 0.040 w2 = 23.684 | F52** |
| **M7** | 3 | -404.536086 | 6.85 | p = 0.005 q = 0.007 | Not Allowed |
| **M8** | 5 | -397.779121 | 9.62 | p0 = 0.960 p1 = 0.040 p = 0.014 q = 0.024 w = 23.935 | F52** |
| **20343633** | **p** | **lnL** | **Kappa** | **Estimates of Parameters** | **Positively Selected Sites** |
| **M0** | 11 | -4789.631059 | 2.85 | w = 0.601 | None |
| **M1** | 3 | -4754.985284 | 2.72 | p0 = 0.522 p1 = 0.478 | Not allowed |
| **M2** | 5 | -4746.197601 | 3.00 | p0 = 0.664 p1 = 0.119 p2 = 0.218 w2 = 2.421 | D236** |
| **M7** | 3 | -4755.150759 | 2.75 | p = 0.005 q = 0.005 | Not Allowed |
| **M8** | 5 | -4746.203341 | 3.00 | p0 = 0.749 p1 = 0.251 p = 0.507 q = 2.670 w = 2.326 | R74* G220* G232* D236** L238* Q285* A300* T356* C381* K400* F406* F407* G503* |
| **20439293** | **p** | **lnL** | **Kappa** | **Estimates of Parameters** | **Positively Selected Sites** |
| **M0** | 5 | -3614.956392 | 3.16 | w = 0.824 | None |
| **M1** | 3 | -3609.603219 | 2.78 | p0 = 0.539 p1 = 0.461 | Not allowed |
| **M2** | 5 | -3593.132008 | 3.57 | p0 = 0.912 p1 = 0.000 p2 = 0.088 w2 = 12.262 | G88* E234* S240* E245* H246** G256* N290* R336* K358* |
| **M7** | 3 | -3609.667338 | 2.84 | p = 0.005 q = 0.005 | Not Allowed |
| **M8** | 5 | -3593.132005 | 3.57 | p0 = 0.912 p1 = 0.088 p = 0.005 q = 99.000 w = 12.262 | G88* E234* S240* E245* H246** G256* N290* R336* K358* |
| **20461581** | **p** | **lnL** | **Kappa** | **Estimates of Parameters** | **Positively Selected Sites** |
| **M0** | 9 | -2585.211582 | 2.07 | w = 0.783 | None |
| **M1** | 3 | -2577.746654 | 1.72 | p0 = 0.645 p1 = 0.355 | Not allowed |
| **M2** | 5 | -2558.676606 | 2.17 | p0 = 0.596 p1 = 0.373 p2 = 0.031 w2 = 34.540 | E42* G92* L93** R97* E98* Y99** R101* I102* K117** V221* Q384* |
| **M7** | 3 | -2577.856953 | 1.77 | p = 0.005 q = 0.008 | Not Allowed |
| **M8** | 5 | -2558.678473 | 2.17 | p0 = 0.969 p1 = 0.031 p = 0.011 q = 0.019 w = 35.122 | E42* G92* L93** R97* E98** Y99** R101** I102* K117** V221* Q384* |
| **20529562** | **p** | **lnL** | **Kappa** | **Estimates of Parameters** | **Positively Selected Sites** |
| **M0** | 7 | -5901.816522 | 2.49 | w = 0.804 | None |
| **M1** | 3 | -5824.720227 | 2.21 | p0 = 0.584 p1 = 0.416 | Not allowed |
| **M2** | 5 | -5746.177947 | 2.86 | p0 = 0.514 p1 = 0.418 p2 = 0.068 w2 = 10.351 | R82** C83** E116* D117* A156* R199** R200** L259* G260** H261** T262** G266* E267** E299* T341** G343* K363* F364* P365* D370** S377** K378* R390* N391** A392** Q393** G398* P400** V401** Y402** A403** H419** S426* R428** G522* A525** V531** I756* R768* R839* |
| **M7** | 3 | -5824.875548 | 2.20 | p = 0.005 q = 0.007 | Not Allowed |
| **M8** | 5 | -5746.282950 | 2.85 | p0 = 0.930 p1 = 0.070 p = 0.022 q = 0.028 w = 10.117 | R82** C83** E116* D117* A156* R199** R200** L259* G260** H261** T262** G266* E267** E299** T341** G343* R361* K363* F364** P365** D370** S377** K378* R390* N391** A392** Q393** D397* G398** P400** V401** Y402** A403** H419** S426* R428** G522* A525** V531** I756** R768* R838* R839* |
| **20975481** | **p** | **lnL** | **Kappa** | **Estimates of Parameters** | **Positively Selected Sites** |
| **M0** | 5 | -2139.679421 | 1.59 | w = 0.687 | None |
| **M1** | 3 | -2135.542432 | 1.41 | p0 = 0.545 p1 = 0.455 | Not allowed |
| **M2** | 5 | -2126.362019 | 1.90 | p0 = 0.951 p1 = 0.000 p2 = 0.049 w2 = 17.682 | F23* H26** Q32* E421* |
| **M7** | 3 | -2135.616760 | 1.45 | p = 0.005 q = 0.005 | Not Allowed |
| **M8** | 5 | -2126.362040 | 1.90 | p0 = 0.951 p1 = 0.049 p = 57.629 q = 99.000 w = 17.686 | I22* F23* H26** Q32** T42* E421* D423* |
| **21139502** | **p** | **lnL** | **Kappa** | **Estimates of Parameters** | **Positively Selected Sites** |
| **M0** | 9 | -7394.891394 | 2.02 | w = 0.827 | None |
| **M1** | 3 | -7300.926719 | 1.68 | p0 = 0.702 p1 = 0.298 | Not allowed |
| **M2** | 5 | -7160.985868 | 2.03 | p0 = 0.831 p1 = 0.067 p2 = 0.101 w2 = 12.449 | None |
| **M7** | 3 | -7300.930655 | 1.68 | p = 0.005 q = 0.012 | Not Allowed |
| **M8** | 5 | -7161.055595 | 2.03 | p0 = 0.904 p1 = 0.096 p = 0.005 q = 0.051 w = 12.949 | None |
| **21586974** | **p** | **lnL** | **Kappa** | **Estimates of Parameters** | **Positively Selected Sites** |
| **M0** | 17 | -874.010280 | 6.86 | w = 1.074 | None |
| **M1** | 3 | -867.657551 | 6.05 | p0 = 0.491 p1 = 0.509 | Not allowed |
| **M2** | 5 | -859.346138 | 6.82 | p0 = 0.510 p1 = 0.350 p2 = 0.140 w2 = 5.928 | A7** V70** T78* |
| **M7** | 3 | -867.662023 | 6.03 | p = 0.005 q = 0.005 | Not Allowed |
| **M8** | 5 | -859.346663 | 6.82 | p0 = 0.857 p1 = 0.143 p = 0.005 q = 0.008 w = 5.857 | A7** S44* V70** T78* R105* |
| **21719250** | **p** | **lnL** | **Kappa** | **Estimates of Parameters** | **Positively Selected Sites** |
| **M0** | 9 | -2224.862856 | 1.96 | w = 0.792 | None |
| **M1** | 3 | -2212.007295 | 1.64 | p0 = 0.618 p1 = 0.382 | Not allowed |
| **M2** | 5 | -2189.258123 | 1.87 | p0 = 0.884 p1 = 0.000 p2 = 0.116 w2 = 12.678 | D151* T170* R174* K188* N195* D315* |
| **M7** | 3 | -2212.038789 | 1.66 | p = 0.005 q = 0.008 | Not Allowed |
| **M8** | 5 | -2189.258168 | 1.87 | p0 = 0.884 p1 = 0.116 p = 5.868 q = 99.000 w = 12.678 | V163* T170* |
| **21769349** | **p** | **lnL** | **Kappa** | **Estimates of Parameters** | **Positively Selected Sites** |
| **M0** | 5 | -1182.578759 | 1.05 | w = 0.576 | None |
| **M1** | 3 | -1173.639208 | 0.96 | p0 = 0.558 p1 = 0.442 | Not allowed |
| **M2** | 5 | -1165.548540 | 1.20 | p0 = 0.912 p1 = 0.000 p2 = 0.088 w2 = 10.587 | K113* R187* E188* Q198* Q199* T200* A207* |
| **M7** | 3 | -1173.796209 | 0.94 | p = 0.005 q = 0.007 | Not Allowed |
| **M8** | 5 | -1165.551889 | 1.20 | p0 = 0.912 p1 = 0.088 p = 45.215 q = 99.000 w = 10.597 | A111* K113* Y122* R187* E188** Q198* Q199* T200* A207* |
| **21943524** | **p** | **lnL** | **Kappa** | **Estimates of Parameters** | **Positively Selected Sites** |
| **M0** | 15 | -11123.634146 | 2.64 | w = 0.598 | None |
| **M1** | 3 | -10896.714657 | 2.48 | p0 = 0.613 p1 = 0.387 | Not allowed |
| **M2** | 5 | -10805.070074 | 2.82 | p0 = 0.593 p1 = 0.346 p2 = 0.061 w2 = 5.655 | I43* A63** E78** D81** D82** L87* G133* W149** V255** G277** S278** I279** G305** P306** L307* S356** Q365* A376* G847* I849* S851* G852* G865* P874* G1070* S1071* T1072* Q1152* T1219* S1222* S1247* R1255* A1260** |
| **M7** | 3 | -10896.992579 | 2.49 | p = 0.005 q = 0.008 | Not Allowed |
| **M8** | 5 | -10805.841775 | 2.83 | p0 = 0.946 p1 = 0.054 p = 0.005 q = 0.008 w = 6.061 | I43* A63** E78** D81** D82** L87* H126* G133** W149** V255** G277** S278** I279** G305** P306** L307* S356** G363* Q365** N375* A376* K442* G847** I849** S851* G852* R855* G865* I866* P874** R933* D943* G1070** S1071* T1072* K1125* Q1152** T1219** S1222** L1226* S1247** R1255** A1260** D1261* |
| **21995461** | **p** | **lnL** | **Kappa** | **Estimates of Parameters** | **Positively Selected Sites** |
| **M0** | 5 | -3896.712150 | 1.30 | w = 0.547 | None |
| **M1** | 3 | -3867.368419 | 1.01 | p0 = 0.788 p1 = 0.212 | Not allowed |
| **M2** | 5 | -3814.361956 | 1.26 | p0 = 0.963 p1 = 0.000 p2 = 0.037 w2 = 33.603 | V118* |
| **M7** | 3 | -3867.415820 | 1.00 | p = 0.005 q = 0.020 | Not Allowed |
| **M8** | 5 | -3814.376805 | 1.25 | p0 = 0.963 p1 = 0.037 p = 0.009 q = 0.162 w = 33.458 | V118* |
| **22063411** | **p** | **lnL** | **Kappa** | **Estimates of Parameters** | **Positively Selected Sites** |
| **M0** | 5 | -2427.373667 | 1.92 | w = 0.600 | None |
| **M1** | 3 | -2420.296543 | 1.83 | p0 = 0.584 p1 = 0.416 | Not allowed |
| **M2** | 5 | -2408.694563 | 2.17 | p0 = 0.870 p1 = 0.021 p2 = 0.110 w2 = 8.278 | P155* L157* N167* A172* Q175* T179* I295* D341* V367* |
| **M7** | 3 | -2420.315063 | 1.82 | p = 0.005 q = 0.008 | Not Allowed |
| **M8** | 5 | -2408.694785 | 2.17 | p0 = 0.887 p1 = 0.113 p = 0.006 q = 0.132 w = 8.144 | P155* L157* T160* N167* A172* Q175* T179* I295* D341* V367* |
